# Proposal of the Annotation of Phosphorylated Amino Acids and Peptides Using Biological and Chemical Codes

**DOI:** 10.3390/molecules26030712

**Published:** 2021-01-29

**Authors:** Piotr Minkiewicz, Małgorzata Darewicz, Anna Iwaniak, Marta Turło

**Affiliations:** Department of Food Biochemistry, University of Warmia and Mazury in Olsztyn, Plac Cieszyński 1, 10-726 Olsztyn-Kortowo, Poland; darewicz@uwm.edu.pl (M.D.); ami@uwm.edu.pl (A.I.); marta.turlo@uwm.edu.pl (M.T.)

**Keywords:** amino acids, peptides, phosphorylation, phosphate groups, databases, code, bioinformatics, cheminformatics, SMILES

## Abstract

Phosphorylation represents one of the most important modifications of amino acids, peptides, and proteins. By modifying the latter, it is useful in improving the functional properties of foods. Although all these substances are broadly annotated in internet databases, there is no unified code for their annotation. The present publication aims to describe a simple code for the annotation of phosphopeptide sequences. The proposed code describes the location of phosphate residues in amino acid side chains (including new rules of atom numbering in amino acids) and the diversity of phosphate residues (e.g., di- and triphosphate residues and phosphate amidation). This article also includes translating the proposed biological code into SMILES, being the most commonly used chemical code. Finally, it discusses possible errors associated with applying the proposed code and in the resulting SMILES representations of phosphopeptides. The proposed code can be extended to describe other modifications in the future.

## 1. Introduction

Phosphorylation belongs to the most important modifications of amino acid residues in peptides and proteins [1,2]. According to Li et al. [3], phosphorylation of food proteins is a useful method for improving their functional properties. Some food products containing proteins (like, e.g., milk, yogurt, or cheeses) can also be sources of phosphopeptides that affect many body functions [4].

The most typical phosphorylation sites in peptides and proteins are serine, threonine, and tyrosine residues. Other residues susceptible to this modification are hydroxylysine, hydroxyproline, lysine, arginine, histidine tryptophan, aspartic acid, glutamic acid, and cysteine [1,5,6,7,8,9,10]. The N-terminal phosphorylation has not been found in proteins but is possible in peptides [10].

Biologically active peptides, including those derived from food sources, are annotated in many databases available via the Internet. Some software types utilize peptide information as an input [11,12,13,14] and use two kinds of languages for peptide annotation—biological and chemical [15,16]. The biological languages (also named residue-based notations [17]) describe large biomolecules composed of repeatable units, like, e.g., amino acid residues annotated with the one-letter and multi-letter code that serve for peptide annotation.

In turn, the chemical languages (atom-based notations) serve mainly to reflect the chemical diversity of small molecules and enable annotating individual atoms in molecules. SMILES [18] is the most commonly used chemical language. Other chemical languages include SYBYL Line Notation (SLN) [19,20] and InChI [21]. 

Amino acid sequences written in a one-letter code are utilized in specialized peptide databases such as EROP-Moscow [22], PepBank [23], or BIOPEP-UWM [24]. The BRENDA database of enzymes [25] and the Norine database of non-ribosomal peptides [26] utilize a multi-letter code for amino acid description. In turn, the SATPdb database [27] utilizes a mixed code—protein amino acids and their D-enantiomers are annotated using a one-letter code, whereas non-protein amino acids—using a multi-letter code. All databases and other bioinformatic and cheminformatic tools cited in this paper are summarized in Table 1. The HELM notation [28,29] designed as a universal language for biopolymer description or LINUCS [30] designed for oligosaccharide description are also used to annotate peptides. The chemical languages are used to annotate peptides in chemical databases, such as PubChem, ChemSpider, and ChEMBL. Codes used for peptide annotation have been recently discussed by David et al. [31].

There is no standardized and commonly accepted biological code enabling the annotation of sequences containing modified (e.g., phosphorylated) amino acid residues. The simplest way is to write an amino acid sequence in a one-letter code and complete the information about the modification in the comments. In texts designed as human-readable, amino acid residues containing the phosphate group may be highlighted in sequences (underlined, displayed using color or bold fonts). Recent examples of this way of phosphate annotation may be found in the articles published by Savastano et al. [41,42], Pourjoula et al. [43], and Bekker-Jensen et al. [44]. The phosphorylation of amino acid residues is recently annotated using the letter “p” before one letter symbol of the amino acid [45,46,47,48]. This notation is applied mainly to describe the results of proteomic experiments. It is very simple (e.g., compared with HELM), compact, and easily human-readable. The above notation of peptides provides information about phosphorylation together with an amino acid sequence. However, this type of annotating phosphorylation has some severe limitations. The symbol “p” may mean phosphorylation or amino acid D-proline. The second opportunity is utilized, e.g., in peptide databases such as BIOPEP-UWM or SATPdb.

A machine-readable code describing the molecular diversity of peptides should discriminate between one-letter symbols of amino acids and symbols of post-translational modifications. The annotation of phosphate groups using the symbol “*” (for instance, S* indicating phosphoserine) fulfills this recommendation and has been used for many years [49,50]. Moreover, a database of phosphopeptides should be easily screened using unmodified amino acid sequences as a query. For instance, the BIOPEP-UWM database [24] has an option named “profile of potential biological activity” that enables finding bioactive fragments in any protein sequence taken from, e.g., the UniProt database. This type of search should also be feasible for phosphopeptides.

The present work aims to propose a code for phosphopeptide annotation with the following features:

- Enabling the description of the diversity of phosphorylation sites and phosphate groups naturally occurring in amino acid and peptide molecules;
- Maintaining the balance between human and machine readability;
- Compatible with a standard one-letter code of amino acid sequences;
- Enabling easy conversion into the chemical code SMILES;
- Enabling future development aimed at the annotation of other amino acid modifications apart from phosphorylation.

## 2. Annotation of Amino Acids and Phosphate Groups

Sequences of peptides consisting of 20 common proteinogenic amino acids, selenocysteine, pyrrolysine, and their D-enantiomers are encoded using a standard one-letter code (“A” and “a” for alanine and D-alanine, respectively; “C” and “c” for cysteine and D-cysteine, respectively, etc.). Non-proteinogenic and unnatural amino acids are usually annotated using a multi-letter code. It has been developed based on a three-letter code of protein amino acids. 

Our proposal includes a multi-letter abbreviation written between “<” and “>” characters. In the case of hydroxyproline and hydroxylysine, the hydroxylation makes the carbon atom asymmetric. Its configuration is indicated by the symbol (R)—rectus or (S)—sinister (see Table 2 in the main text and Tables S1 and S2 in the Supplementary Materials). Text or other symbols in such parentheses should be considered equivalent to a one-letter symbol of a protein amino acid. 

The code for the well-known amino acids utilizes abbreviations of their common names. This rule cannot be considered as obligatory. The entire chemical space of small molecules contains hundreds of billions of stable compounds containing up to 17 atoms [51]. The entire subspace of amino acids (all possible amino acids understood as components containing at least one carboxyl and at least one amine group) also should be very large. We can expect that more and more amino acids will be discovered and synthesized in the future. Incorporating unnatural amino acids into peptide and protein sequences is the object of intensive investigations [52,53,54]. 

Abbreviations used to describe amino acids should enable their unambiguous description and provide some information about compound structure, if necessary and possible. They may play a role similar to InChIKeys in chemical information [21]. The main feature of the biological codes is their compactness [16]. Abbreviations used to annotate non-proteinogenic and unnatural amino acids should be short (ultimately: shorter than SMILES or other chemical representations). Hydroxyproline and hydroxylysine annotations (Table 2 in the main text and Table S1 in Supplementary Materials) are examples of attempts to fulfill the above recommendations. 

Amino acid symbols may include Latin letters, numbers, Greek letters, and any other characters. The “ΔF” symbol of didehydrophenylalanine (PubChem CID: 17902612), used in the SATPdb database (or the <ΔF> symbol according to the convention proposed in this article), may serve as an example of using Greek letters for amino acid annotation. Abbreviations of systematic (IUPAC) names of amino acids may also be used due to their major advantage. They may be generated automatically by computer software. The vocabulary of amino acid multi-letter symbols should include traditional abbreviations used to date in literature and such databases as SwissSidechain, Norine, and SATPdb. All biological and chemical representations of amino acids should be easily applicable in search engines. Abbreviations used to describe other classes of compounds (e.g., abbreviations of monosaccharides used in one of the many existing formats for carbohydrates and carbohydrate moieties annotation [17,55,56,57]) should be avoided if possible. The proposal of this restriction is justified because using the same abbreviation to describe compounds from various classes may appear confusing and lead to errors in computer programs, as pointed out in our previous paper [58] on the example of amino acid and nucleotide sequences. The symbols proposed to encode phosphopeptides are summarized in Table 2. 

There is no unique system for annotating modifications of amino acid residues in sequences. Parentheses “( )“ are used to indicate modifications [50,59]. We propose writing symbols of modifications in the following brackets “[…]”. The IUPAC recommends these brackets for annotating sugar residues in glycopeptides. Examples of such annotations are presented at the NCBI Glycans website. It seems to be logical to apply the same notation to present other modifications, e.g., phosphorylation. Amidation of phosphate groups is indicated with the symbol “~” (see Table 2 in the main text and Table S1 in the Supplementary Materials) used to date in the BIOPEP-UWM database to annotate C-terminal amidation [24]. The amidation of C-terminal carboxyl groups of peptides is indicated in the BIOPEP-UWM database with the symbol without parentheses. The same convention is proposed for the amide group modifying the terminal phosphate group or connecting two phosphate groups. The symbol of amide group linked to the non-terminal phosphate group is written in parentheses (see Table 2 in the main text and Table S1 in the Supplementary Materials).

## 3. Location of Modifications in Amino Acid Residues

The numbering of atoms being the potential modification sites in amino acid residues is presented in Table 3. In some cases (hydroxyproline isomers, histidine), there is more than one phosphorylation site [5,7,8]. In most of the proteinogenic amino acids, the numbering of carbon atoms is univocal and follows the rules designed for carboxyl acids where the carbon atom in the α-carboxyl group possesses No. 1.

The same rule is continuously applied to all amino acids mentioned in this article. Such numbering may appear controversial in the case of amino acids containing rings (tyrosine, hydroxyproline, histidine, tryptophan). Atom numbering according to IUPAC recommendations and used in, e.g., PubChem database includes separate numbering for chains and rings, especially heterocyclic ones. For instance, in histidine (PubChem CID 6274), a systematic name presented in the databases assigns number 1 to two atoms—a carbon atom in a carboxyl group (according to the rules designed to describe carboxyl acids) and one of the nitrogen atoms within the ring (according to the rules designed for the description of heterocyclic compounds).

Such numbering seems confusing from the viewpoint of designing a machine-readable biological code, although it is easily human-readable due to tradition. Continuous atom numbering in amino acid residues enables the unambiguous location of any modification (not only phosphorylation) in the amino acid residue. Our proposal mimics atom numbering in sugar residues. Formats for the annotation of carbohydrates and carbohydrate moieties [17,55,56,57] use names and abbreviations assigning No. 1 to hemiacetal or acetal carbon atom, whereas the notation used in general chemical databases (e.g., PubChem) assigns No. 1 to the oxygen atom, considering a sugar molecule as a heterocyclic compound. The INChI code [21] and the ReactionCode [60] also contain unambiguous numbering of atoms in a molecule. The rules of atom numbering proposed below seem to be more intuitive than these used in the above codes.

**Figure 1:**
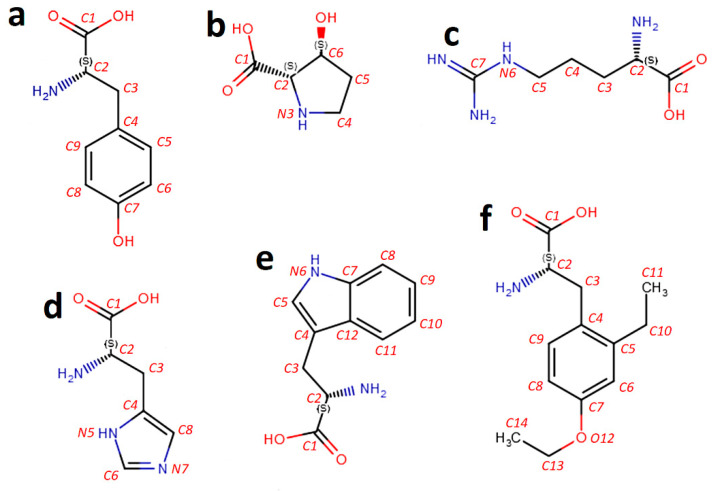
Examples of the continuous numbering of atoms in amino acid molecules: (**a**) tyrosine ((2*S*)-2-amino-3-(4-hydroxyphenyl)propanoic acid); (**b**) 3-hydroxyproline ((2*S*,3*S*)-3-hydroxypyrrolidine-2-carboxylic acid); (**c**) arginine ((2*S*)-2-amino-5-carbamimidamidopentanoic acid); (**d**) histidine ((2*S*)-2-amino-3-(1H-imidazol-5-yl)propanoic acid); (**e**) tryptophan ((2*S*)-2-amino-3-(1*H*-indol-3-yl)propanoic acid); (**f**) (2*S*)-2-amino-3-(4-ethoxy-2-ethylphenyl)propanoic acid. Figure prepared using Marvin Sketch editor (Chem Axon, Budapest, Hungary).

Examples of numbering atoms in amino acid molecules are presented in Figure 1. Atoms in amino acid residues are numbered according to the following rules:

1. Carbon atom within the carboxylic group receives No. 1 (like in the IUPAC names of carboxylic acids).
2. If there are more carboxyl groups, the nearest from the amine group receives No. 1;
3. Atoms other than carbon (N, O, S, etc.) receive numbers only if they are not terminal. (Atoms in groups: -NH-; -N=; -O-; -S- possess numbers, whereas atoms in groups -NH_2_; -OH; -SH—not).
4. Atoms in the rings are numbered according to the rule of the smallest sum of digits, including rules 1–3. The atom connected with a substituent containing the carboxyl group No. 1 possesses priority over heteroatoms and other substituents.
5. Atoms within the ring have priority over atoms in substituents (except for the substituent containing the carboxyl group No. 1).
6. Atoms in side chains bound to the main chain (a chain containing the carboxyl group with carbon atom No. 1) or to the ring are numbered following the location of these chains (including rule 5).
7. Among different substituents at the same carbon or other atom, priority is established based on the Cahn-Ingold-Prelog rules [61].

All molecules presented in Figure 1 are α-amino acids. All carbon atoms in the carboxyl group have No. 1. Nitrogen atom No. 6 in the arginine residue (Figure 1c), nitrogen atoms within the rings in hydroxyproline (Figure 1b), histidine (Figure 1d), and tryptophan (Figure 1e), as well as the oxygen atom within the ethoxyl group (Figure 1f) are examples illustrating Rule 3.

Examples of potential errors in the proposed biological code are presented in Table 4. The first type of error is indicated by a symbol of amino acid with a phosphate group attached to a carbon atom without any functional group (e.g., carbon atom No. 4 in threonine residue) in a natural peptide representation. Phosphate groups are not attached to such carbon atoms. These atoms may be the sites of the attachment of the phosphoric acid derivative linked via a carbon-phosphorus bond [62]. However, such an amino acid or peptide derivatives, named phosphonoamino acids and phosphonopeptides respectively, should be considered as a separate class of compounds. Most of the known phosphonopeptides contain a phosphone group instead of a C-terminal carboxyl group [62]. Few peptides containing phosphonoalanine [63] were synthesized in a laboratory, but natural peptides containing amino acids with phosphonated side chains remain unknown to date.

Another potential error is the attachment of two phosphate groups to the imidazole ring (Error No. 2). Although two phosphate groups can be attached to the same amino acid residue via different functional groups, nitrogen atoms built into imidazole rings make an exception. Only one nitrogen atom in this ring reveals basic properties and can be phosphorylated. It is impossible to perform the phosphorylation of both nitrogen atoms simultaneously.

Representations of No. 3 and No. 4 are inappropriate because carboxyl and amine groups cannot be simultaneously phosphorylated and involved in peptide bond formation.

## 4. Recommendations Concerning Search Engines

Search engines available in a peptide database using the code described here should include the following options: exact match or search for longer peptides containing the query fragment. Peptide sequences, annotated using the proposed code, should be available for the search using traditional sequences as a query. Shorter fragments (subsequences) should be possible to be found in a particular sequence. Traditional sequences, used in such databases as UniProt [39], PepBank [23], EROP-Moscow [22], or BIOPEP-UWM [24], consist of proteinogenic amino acid symbols, annotated using a one-letter code. The compatibility of peptide representations in the proposed code with traditional sequences may be achieved in two ways. The first one is the double annotation of the same peptide. Representations in the proposed code could serve to convert into chemical codes or calculation of molecular masses and masses of fragment ions to enable the identification with mass spectrometry. Representations consisting of one-letter symbols of proteinogenic amino acids may serve for protein database screening or sequence alignments performed using BLAST [64] or a related algorithm.

**Figure 2:**
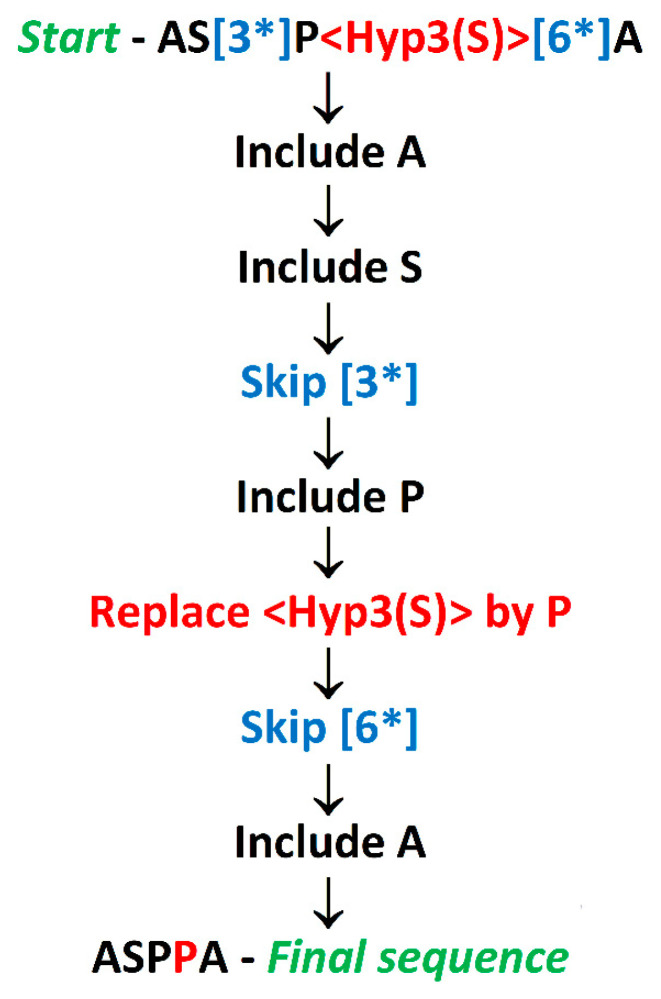
Steps of peptide AS[3*]<Hyp3(S)>[6*]A conversion into an unmodified sequence using rules summarized in Table 5. One-letter symbols of proteinogenic amino acids in the initial peptide representation are presented using black font, modifications not included in the final sequence using blue front, whereas the representation of hydroxyproline and the symbol of a corresponding proline residue using red font.

Among the amino acids mentioned in this article, hydroxyproline isomers and hydroxylysine are products of the post-translational modification (hydroxylation) of proline and lysine, respectively. Symbols of modifications annotated in parentheses […] are not included in unmodified sequences. Operations on particular symbols during the conversion from the code described here into traditional sequences written using a one-letter code are presented in Table 5. The conversion of an exemplary peptide AS[3*]<Hyp3(S)>[6*]A into an unmodified sequence ASPPA is presented in Figure 2. The program working according to the scheme presented in this figure would start from N-terminal residue, leave one-letter symbols of proteinogenic amino acids, skip modification symbols annotated in parentheses, and replace hydroxyproline representation with the proline symbol. The scheme presented in Figure 2 is not applicable if a peptide contains amino acid residues not resulting from the post-translational modifications of the proteinogenic ones (e.g., unnatural amino acids).

## 5. Conversion of the Biological Code into SMILES

The SMILES code [18] is the most popular among the chemical codes and commonly used to annotate peptide structures in chemical databases or as an input for programs predicting and modeling their physicochemical properties and biological activity [65]. SMILES may be easily converted into other chemical codes. An algorithm for the construction of peptide SMILES representations has been described by Siani and co-workers [66]. A simplified version of this algorithm has been applied in such programs as CycloPs [35] and BIOPEP-UWM [24].

SMILES strings of amino acids should be arranged as follows: α-amine group, α-carbon atom, side chain, and α-carboxyl group. This arrangement of α-amino acid representations is used in several bioinformatic and cheminformatic tools, such as Chemical Identifier Resolver [33], CycloPs source code [35], SwissSideChain [38], and BIOPEP-UWM [24]. SMILES strings of amino acids, arranged according to the above rule, may be generated by the Chemical Identifier Resolver program [33]. Representations of proteinogenic amino acids and their d-enantiomers are available in the BIOPEP-UWM database and can be displayed using the “SMILES” application [24]. CycloPs source code in Github (address in Table 1) includes a list of rearranged SMILES strings of unnatural and non-proteinogenic amino acids taken from the ZINC database.

Representations of amino acids can also be rearranged manually. In such a case, careful verification of their correctness is necessary. The simplest way to do that is to translate SMILES codes of a given amino acid into InChIKey using Chemical Identifier Resolver, Marvin Sketch, or other program enabling the conversion between various chemical codes. InChIKey obtained before and after the rearrangement should be the same. Verification of amino acid representations retrieved from databases by, e.g., the confrontation between various resources (e.g., PubChem, ChemSpider, ChEMBL, and ZINC) or displaying and checking the structure using a molecule editor is always recommended [65].

Building peptide SMILES strings is easier if they correspond to non-protonated amine groups and non-dissociated carboxyl groups. SMILES representations that include ions are often presented in databases because they are sufficient to predict their metabolism or biological activity. Aromatic rings may be annotated using two SMILES versions—“Kekule” and “aromatic”. The second one is recommended at the OpenSmiles website as describing true electron distribution. However, the first one is used in the PubChem database and recommended as enabling the construction of a standardized version of the molecule structures [67]. Moreover, some search engines do not accept the aromatic version of conjugated or heterocyclic aromatic rings [68], as is the case with histidine and tryptophan among the proteinogenic amino acids. The list of exemplary SMILES strings of phosphorylated amino acids is presented in Table S1, whereas the list of representations of amino acids without phosphate groups—in Table S2 of the Supplementary Materials.

The insertion of phosphate group representations into amino acid SMILES strings may be done in two ways. The first assumes adding SMILES representations of phosphorylated amino acids (see Table S1 in the Supplementary Materials) to the vocabulary. This is the simplest way and can be recommended to annotate a limited number of phosphorylated amino acids (e.g., peptides containing only proteinogenic amino acids, phosphorylated via hydroxyl groups).

**Figure 3:**
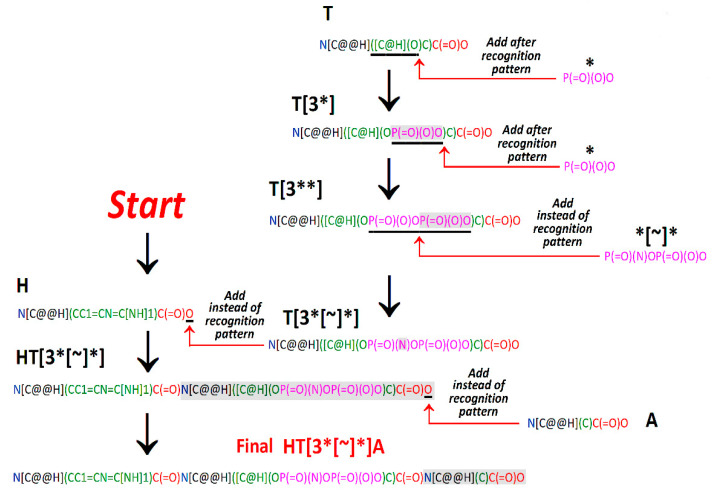
Construction scheme of an exemplary SMILES string of HT[3*[~]*]A—histydyl-threonyl-alanine peptide with a threonine residue modified by the addition of two phosphate groups connected via a phosphodiester bond. The phosphate group linked directly to the amino acid residue is amidated (see Table 2). Color code used in SMILES strings: α-amine group—blue; α-carbon atom—black; side chain—green; phosphate group–pink; α-carboxyl group—red, according to the convention used by Minkiewicz et al. [65] (see Tables S1–S3 in the Supplementary Materials). Recognition patterns in amino acid SMILES strings are underlined, whereas fragments added in a given step are shaded. Terms “After” and “Instead of” mean the location of a new fragment relative to the recognition pattern (see Tables S2 and S3 in the Supplementary Materials). Modification sites are indicated by red arrows.

**Figure 4:**
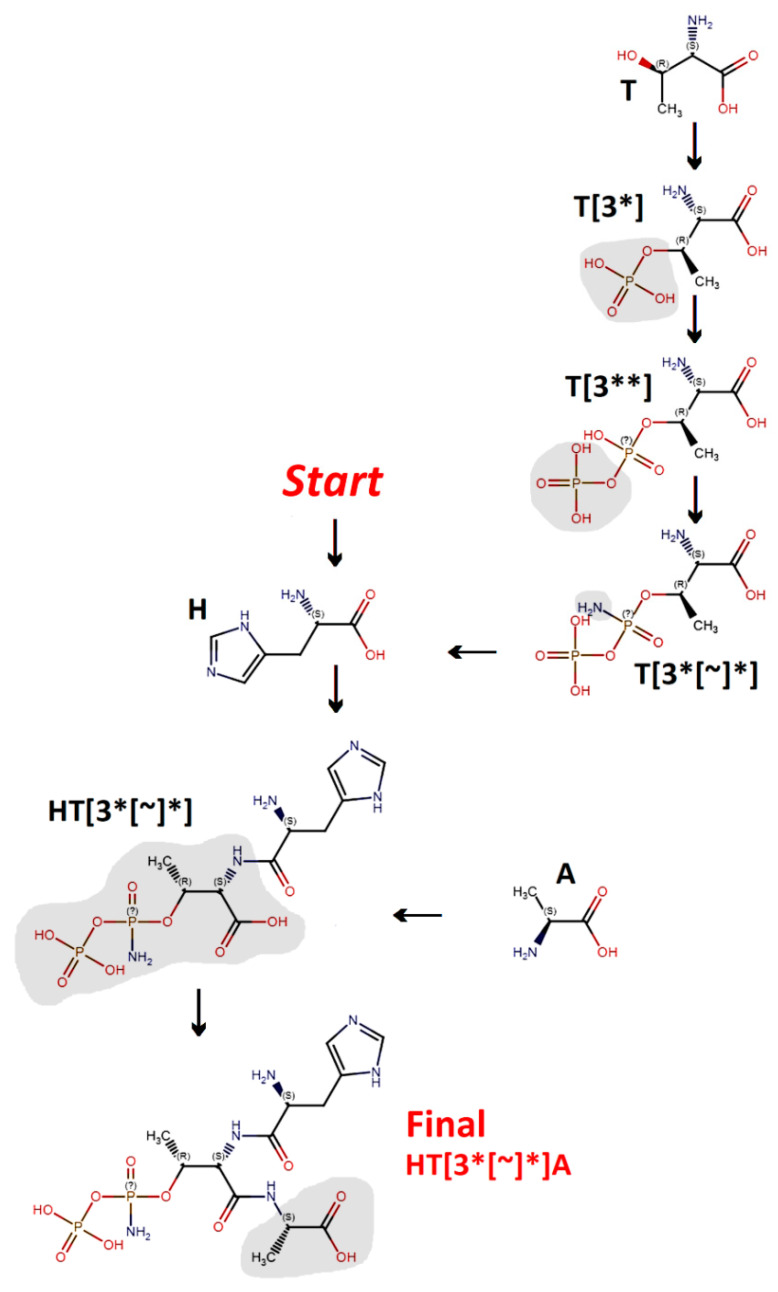
Structures of compounds corresponding to the SMILES strings presented in Figure 3. Fragments added in given steps are shaded. Configuration around asymmetric carbon atoms (R or S) is indicated. Character “?” indicates asymmetric phosphorus atoms without defined configuration. Figure prepared using Marvin Sketch editor.

If the code is intended to be expanded by annotating other modifications, another opportunity can be considered, as illustrated in Figure 3 and Figure 4. Amino acid residues may be considered as scaffolds understood by Arús-Pous et al. [69] as partially-built molecules with defined attachment points. A simple procedure proposed to recognize representations of attachment points in amino acid SMILES strings relies on recognition patterns (Figure 3, Tables S2 and S3). This term mimics the so-called recognition sequences understood as the fragments of polynucleotide sequences recognized by restriction enzymes (endonucleases) [70,71,72].

Here, the recognition pattern is understood as a fragment of the SMILES string attributed to the particular attachment point and enabling its unambiguous recognition. Two attachment points in the same amino acid molecule should not possess the same recognition pattern, but the same pattern may occur in different molecules (see Tables S2 and S3). For instance, the symbol “N” may serve as a recognition pattern if there is only one nitrogen atom in an amino acid molecule. In that case, it indicates the α-amine group. If there are more nitrogen atoms in a molecule—longer patterns are necessary to describe them. The phosphate group representation may be inserted before, after, or instead of the recognition pattern (Tables S2 and S3, Figure 3).

Figure 3 and Figure 4 illustrate the construction of a SMILES representation of HT[3*[~]*]A tripeptide as an example. The construction begins from the N-terminal amino acid residue—histidine (H). Residue No. 2 is modified threonine; its modification should be annotated before its incorporation into the peptide structure. The modification’s insertion starts from the unmodified threonine representation (T). The program constructing the SMILES representation should find the recognition pattern: ([C@H](O corresponding to a hydroxyl group being the attachment point in a threonine string and add phosphate group (*) representation: P(=O)(O)O (shaded in Figure 3 and Figure 4) in the appropriate position (after the recognition pattern). The resulting residue is T[3*]. Phosphate group representation serves as a recognition pattern for the insertion of another phosphate group. It may be done by adding a SMILES string fragment P(=O)(O)O after an identical fragment inserted previously. The resulting residue is T[3**]. The incorporation of an amide group is the next step in modified threonine residue preparation. It is difficult to label unambiguously the oxygen atom in a diphosphate group which should be replaced by a nitrogen atom. Thus, the entire diphosphate group representation: P(=O)(O)OP(=O)(O)O, should be replaced by P(=O)(N)OP(=O)(O)O to obtain a modified threonine residue T[3*[~]*] representation. This representation should be added to the N-terminal histidine string to annotate HT[3*[~]*] Dipeptide. The final step involves the addition of a C-terminal alanine residue (A).

According to our experience [65], the conversion of biological codes into SMILES is a critical step in processing peptide structures. Such programs are often designed and written by interdisciplinary groups including, e.g., chemists, biochemists, and informaticians. The design and validation of codes is a crucial step in the workflow [73].

The manual construction of a set of peptide SMILES strings is the first step of work on the program. A set of manual SMILES strings should be corrected and all details of the procedure should be well explained to achieve the communication between team members representing various specialties. On the other hand, many errors are unavoidable while constructing SMILES representations. This problem has been discussed in our previous publication [65]. Examples of errors in peptide SMILES representations, constructed manually based on the code proposed in this article, are presented in Figures S1–S5 in the Supplementary Materials. The errors include the inappropriate arrangement of amino acid representations, e.g., missed parentheses in SMILES code, leading to errors in side-chain structures and inappropriate connection between amino acid representations leading to the inappropriate structure of peptide bonds. All errors were corrected based on the structures displayed using a molecule editor.

## 6. Final Remarks

The article presents a proposal of a standardized, human- and machine-readable code for annotating phosphopeptides. The code is designed to be used in databases annotating phosphopeptides and programs processing their sequences. The proposed code can be translated into SMILES using the procedure being an extension of the CHUCKLES algorithm. It is more specialized than the existing codes for biomacromolecule description, such as LINUCS or HELM, but simpler than the above codes. The proposed notation includes, e.g., the unambiguous numbering of atoms in amino acid residues. It can be extended to utilize symbols of non-proteinogenic or unnatural amino acid symbols and annotation. The extended code may also be used to annotate other types of post-translational and chemical modifications of peptides in the future.

## Figures and Tables

**Table 1:** Bioinformatic and cheminformatic tools cited in this article.

Name of Database or Software ^1^	Website	Reference
BIOPEP-UWM	http://www.uwm.edu.pl/biochemia/index.php/pl/biopep	[24]
BRENDA	https://www.brenda-enzymes.org/	[25]
ChEMBL	https://www.ebi.ac.uk/chembl/	[32]
Chemical Identifier Resolver	https://cactus.nci.nih.gov/chemical/structure	[33]
ChemSpider	http://www.chemspider.com/	[34]
CycloPs—source code	https://github.com/fergaljd/cyclops	[35]
EROP-Moscow	http://erop.inbi.ras.ru/	[22]
NCBI Glycans	https://www.ncbi.nlm.nih.gov/glycans/index.html	[36]
Norine	https://bioinfo.lifl.fr/norine/	[26]
OpenSmiles	http://opensmiles.org/	Provider: Blue Obelisk Initiative
PepBank	http://pepbank.mgh.harvard.edu/	[23]
PubChem	https://pubchem.ncbi.nlm.nih.gov/	[37]
SATPdb	http://crdd.osdd.net/raghava/satpdb/links.php	[27]
SwissSidechain	https://swisssidechain.ch/	[38]
UniProt	https://www.uniprot.org/	[39]
ZINC	http://zinc15.docking.org/	[40]

^1^ All tools summarized in the table were accessed in November 2020.

**Table 2:** Symbols used to annotate amino acids and phosphate residues.

Symbol ^1,2^	Explanation
A; C; D …	Symbols of proteinogenic amino acids
a; c; d …	Symbols of d-enantiomers of proteinogenic amino acids
<…> (e.g., <Hyp3(S)>; <D-Hyp3(R)>)	Symbols of non-proteinogenic, unnatural, and modified amino acids (Examples: 3-hydroxyproline; 3-d-hydroxyproline
[3*]; [4*]; [5*]…	Symbol “*” means phosphate group, the brackets indicate the start and the end of a compound representation fragment annotating modification. The number indicates modification site in the amino acid residue
[3***]	Example: a chain containing three phosphate residues connected to the amino acid residue via the atom No. 3
[3*~]; [4*~]; [5*~]…	Annotation of amidated phosphate groups; the amide group indicated using the character “~”; modification site in the amino acid residue indicated using a number
S[3*]; T[3*]; <Hyp3>[6*] …	Examples of phosphorylated amino acids: phosphoserine, phosphothreonine, hydroxyproline (phosphorylation via the hydroxyl groups)
S[1*][3*]	Serine residue with two phosphate groups linked via α-carboxyl group and hydroxyl group
S~[3*]	Serine residue with amidated α-carboxyl group and phosphorylated hydroxyl group
S[3**~]	Serine residue modified by the attachment of two phosphate groups connected via a phosphodiester bond; amidation of the terminal phosphate group
S[3*~*]	Serine residue modified by the attachment of two phosphate groups connected via the amide group
S[3*[~]*]	Serine residue modified by the attachment of two phosphate groups connected via a phosphodiester bond. Phosphate group linked directly to the amino acid residue is amidated Amide group is annotated as phosphate group modification
Example of phosphopeptide: AS[3*]<Hyp3(S)>[6*]Ga	Peptide: alanine-phosphoserine-phospho-3-hydroxyproline-glycine-d-alanine

^1^ More details concerning the structure of phosphorylated amino acids may be found in Table S1 in Supplementary Materials. ^2^ The numbering of atoms in amino acid residues is presented in the next section and Table 3.

**Table 3:** Possible location of phosphate groups in amino acid residues.

Amino Acid	Phosphorylation or Other Modification	
	Location ^1^	Annotation
α-Carboxyl group in all amino acids mentioned in this text	Atom C1	[1…]
α-Amine group in all amino acids mentioned in this text; nitrogen atoms in proline and hydroxyproline isomers	Atom C2	[2…]
Serine hydroxyl group	Atom C3	[3…]
Threonine hydroxyl group	Atom C3	[3…]
Tyrosine hydroxyl group	Atom C7	[7…]
3-Hydroxyproline hydroxyl group	Atom C6	[6…]
4-Hydroxyproline hydroxyl group	Atom C5	[5…]
5-Hydroxylysine hydroxyl group	Atom C5	[5…]
Aspartic acid β-carboxyl group	Atom C4	[4…]
Glutamic acid γ-carboxyl group	Atom C5	[5…]
Lysine and hydroxylysine ε-amine group	Atom C6	[6…]
Arginine guanidine group	Atom C7	[7…]
Histidine nitrogen atoms within the imidazole ring	Atom N5 or N7	[5…] or [7…]
Tryptophan indole nitrogen atom	Atom N6	[6…]
Cysteine thiol group	Atom C3	[3…]

^1^ More details concerning the numbering of atoms in the amino acid residue may be found in Table S1 in the Supplementary Materials.

**Table 4:** Examples of possible errors in peptide annotation.

No.	Inappropriate Representation of Peptide	Explanation
1.	AT[4*]G	Inappropriate location of a phosphate group in the threonine residue
2.	AH[5*][7*]G	Two phosphorylated nitrogen atoms in the imidazole ring being part of the histidine residue
3.	AS[1*]G	Carboxyl group simultaneously phosphorylated and involved in the formation of a peptide bond
4.	AS[2*]G	Amine group simultaneously phosphorylated and involved in the formation of a peptide bond

**Table 5:** Conversion of a modified peptide representation into an unmodified sequence consisting of proteinogenic amino acids.

Symbol	Operation
One-letter symbol of proteinogenic amino acid	Include into unmodified sequence.
Symbol of modification: “[…]”	Skip during building unmodified sequence.
Symbols of l-hydroxyproline isomers: “<Hyp3(*S*)>; <Hyp4(*R*)>”	Replace by symbol of proline “P”.
Symbol of l-hydroxylysine: <Hyl5(*R*)>	Replace by symbol of lysine “K”.

[…]—Brackets indicate any modification of amino acid residue. For details see Table 2.

## Data Availability

The data presented in this study are available in the article or Supplementary Materials (Tables S1–S3, Figures S1–S5).

## Supplementary Materials

The following are available online, Tables S1–S3, Figures S1–S5.

## Abbreviations

*	Proposed symbol of phosphate group
~	Proposed symbol of amidation
BLAST	Basic Local Alignment Search Tool
BRENDA	Braunschweig Enzyme Database
[C@] and [C@@]	Symbols of chiral carbon atoms in SMILES code
CID	Compound Identifier (in PubChem database)
ΔF	Symbol of dehydrophenylalanine according to the SATPdb database
EMBL	European Molecular Biology Laboratory
EROP-Moscow	Endogenous Regulatory OligoPeptide knowledgebase-Moscow
HELM	Hierarchical Editing Language for Macromolecules
Hyl	Hydoxylysine
Hyp	Hydroxyproline
InChI	International Chemical Identifier
InChIKey	Key of International Chemical Identifier
IUPAC	International Union of Pure and Applied Chemistry
LINUCS	LInear Notation for Unique description of Carbohydrate Sequences
NCBI	National Center for Biotechnology Information
P	Depending on the context, one-letter symbol of proline or phosphorus symbol
p	Depending on context, one-letter symbol of D-proline or symbol of phosphorylation of amino acid residue
R	One-letter symbol of arginine
(R)	Proposed symbol of configuration of substituents around asymmetric atom: “*Rectus*”
S	One-letter symbol of serine
(S)	Proposed symbol of configuration of substituents around asymmetric atom: “*Sinister*”
SATPdb	Structurally Annotated Therapeutic Peptides database
SLN	SYBYL Line Notation
SMILES	Simplified Molecular Input Line Entry System or Simplified Molecular Input Line Entry Specification
UWM	University of Warmia and Mazury in Olsztyn, Poland
