# Supplementary material for: Proposal of the Annotation of Phosphorylated Amino Acids and Peptides Using Biological and Chemical Codes
Source: Molecules. 2021 Jan 29;26(3):712. doi: 10.3390/molecules26030712 (PMC7866520; doi:10.3390/molecules26030712)
Supplement: Supplementary file 1 [file molecules-26-00712-s001.pdf]

Proposal of of the Annotation of Phosphorylated Amino Acids and Peptides Using Biological and Chemical Codes

Piotr Minkiewicz, Małgorzata Darewicz, Anna Iwaniak and Marta Turło,

Supplement

Table S1. Proposed codes, structures, and SMILES representations of the examples of phosphorylated amino acids (with focus on naturally occurring compounds).

Name	Code	Structure[1]	SMILES[1; 2; 3]	PubChem CID
O-phospho-L-serine	S[3*]	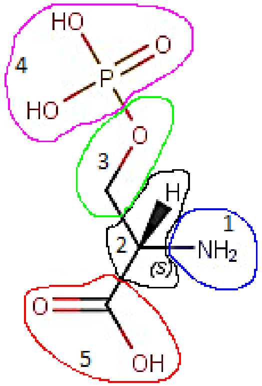	N[C@@H](COP(=O)(O)O)C(=O)O	68841
O-phospho-D-serine	s[3*]	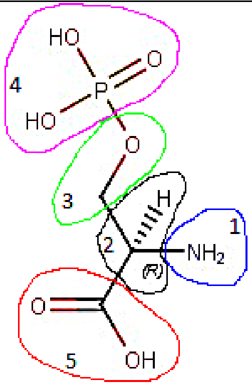	N[C@H](COP(=O)(O)O)C(=O)O	439747

O-phospho-L-threonine	T[3*]	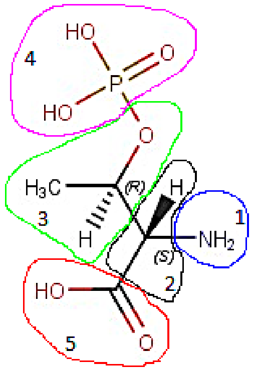	N[C@@H]([C@H](OP(=O)(O)O)C)C(=O)O	3246323
O-phospho-D-threonine	t[3*]	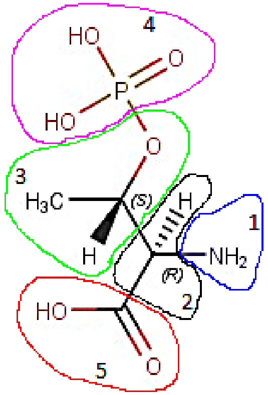	N[C@H]([C@@H](OP(=O)(O)O)C)C(=O)O	10976469

O-phospho-L-tyrosine	Y[7*]	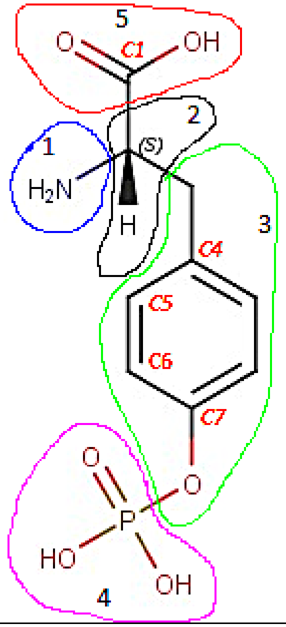	N[C@@H](CC1=CC=C(C=C1)OP(=O)(O)O)C(=O)O	30819
O-phospho-D-tyrosine	y[7*]	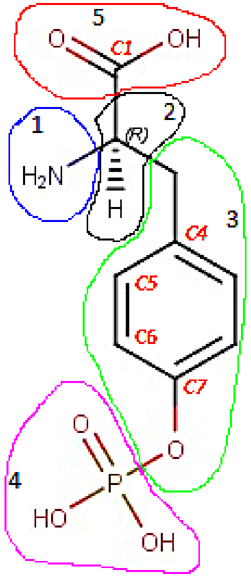	N[C@H](CC1=CC=C(C=C1)OP(=O)(O)O)C(=O)O	40520294

(3S)-3-(Phosphonooxy)-L-proline (phosphorylated 3-hydroxy-L-proline)	<Hyp3(S)>[6*]	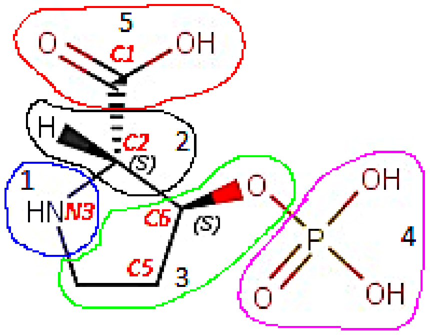	N1[C@@H]([C@@H](OP(=O)(O)O)CC1)C(=O)O	-
(3R)-3-(Phosphonooxy)-D-proline (phosphorylated 3-hydroxy-D-proline)	<D-Hyp3(R)>[6*]	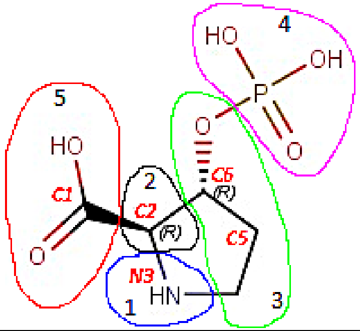	N1[C@H]([C@H](OP(=O)(O)O)CC1)C(=O)O	-
(4R)-4-(Phosphonooxy)-L-proline (phosphorylated 4-hydroxy-L-proline)	<Hyp4(R)>[5*]	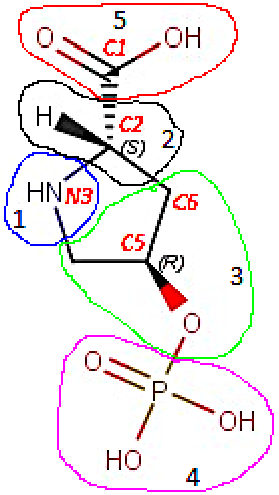	N1[C@@H](C[C@@H](OP(=O)(O)O)C1)C(=O)O	189116

(4S)-4-(Phosphonooxy)-D-proline (phosphorylated 4-hydroxy-D-proline)	<D-Hyp4(S)>[5*]	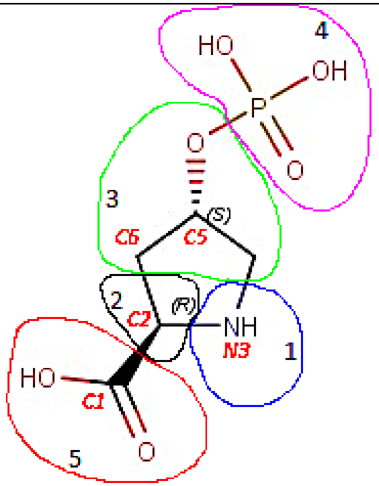	N1[C@H](C[C@H](OP(=O)(O)O)C1)C(=O)O	15677507
Phosphohydroxyproline	<Hyp>[*]	No defined structure due to lack of information about location of hydroxyl group	No defined SMILES string	
(5R)-5-phosphonooxy-L-lysine	<Hyl5(R)>[5*]	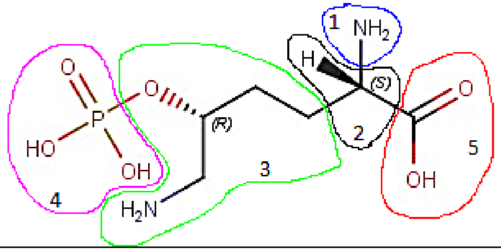	N[C@@H](CC[C@@H](OP(=O)(O)O)CN)C(=O)O	25163995
(5S)-5-phosphonooxy-D-lysine	<D-Hyl5(S)>[5*]	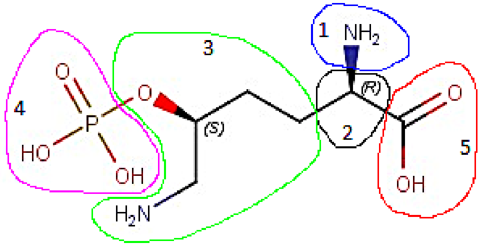	N[C@H](CC[C@H](OP(=O)(O)O)CN)C(=O)O	-

Aspartyl phosphate (beta-aspartyl phosphate)	D[4*]	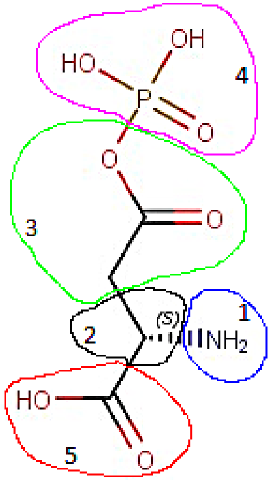	N[C@@H](CC(=O)OP(=O)(O)O)C(=O)O	152441
D-aspartyl phosphate (beta-D-aspartyl phosphate)	d[4*]	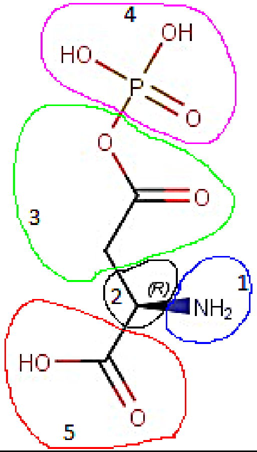	N[C@H](CC(=O)OP(=O)(O)O)C(=O)O	92209504
Glutamyl phosphate (gamma-glutamyl phosphate)	E[5*]	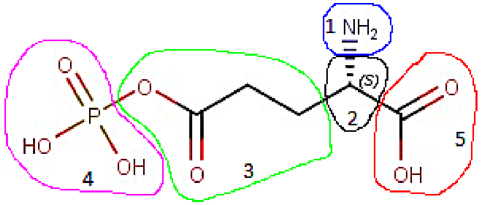	N[C@@H](CCC(=O)OP(=O)(O)O)C(=O)O	193475

D-Glutamyl phosphate (gamma-D-glutamyl phosphate)	e[5*]	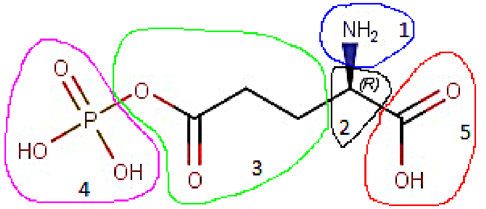	N[C@H](CCC(=O)OP(=O)(O)O)C(=O)O	24820755
N(6)-Phosphono-L-lysine<br>N-epsilon-Phospholysine	K[6*]	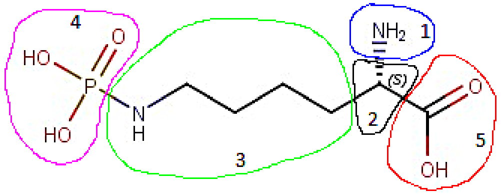	N[C@@H](CCCCNP(=O)(O)O)C(=O)O	161086
N(6)-Phosphono-D-lysine<br>N-epsilon-Phospho-D-lysine	k[6*]	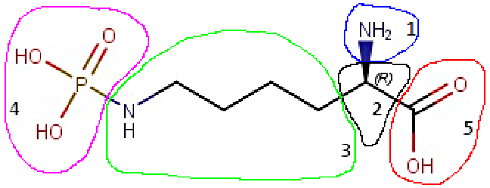	N[C@H](CCCCNP(=O)(O)O)C(=O)O	-
(5R)-N(6)-phosphonooxy-L-lysine<br>N-epsilon-Phospho-5-hydroxy-L-lysine	<Hyl5(R)>[6*]	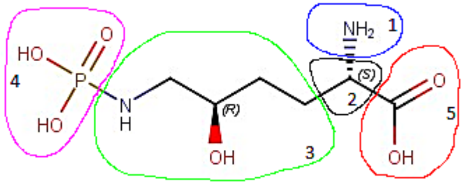	N[C@@H](CC[C@@H](O)CNP(=O)(O)O)C(=O)O	-
(5S)-N(6)-phosphonooxy-D-lysine<br>N-epsilon-Phospho-5-hydroxy-D-lysine	<D-Hyl5(S)>[6*]	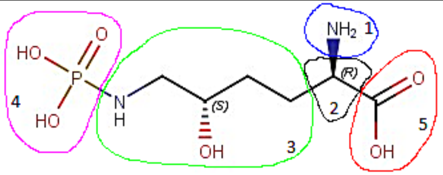	N[C@H](CC[C@H](O)CNP(=O)(O)O)C(=O)O	-
N-(omega)-Phospho-L-arginine	R[7*]	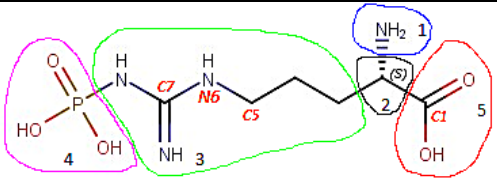	N[C@@H](CCCNC(=N)NP(=O)(O)O)C(=O)O	92150

N-(omega)-Phospho-D-arginine	r[7*]	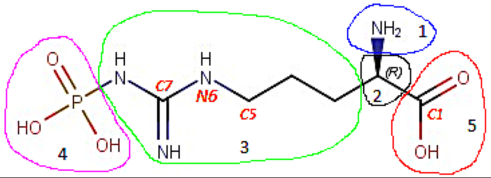	N[C@H](CCCNC(=N)NP(=O)(O)O)C(=O)O	-
1-Phosphono-L-histidine;<br>1-phosphohistidine	H[7*]	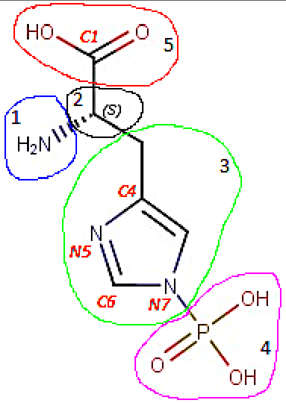	N[C@@H](CC1=C[N](C=N1)P(=O)(O)O)C(=O)O	15458486
1-Phosphono-D-histidine;<br>1-phospho-D-histidine	h[7*]	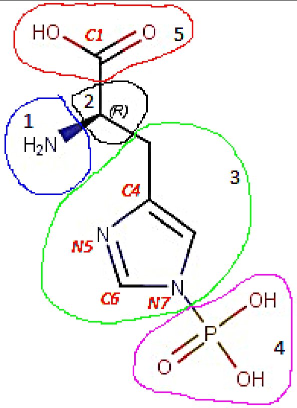	N[C@H](CC1=C[N](C=N1)P(=O)(O)O)C(=O)O	-

3-Phosphono-L-histidine;<br>3-phosphohistidine	H[5*]	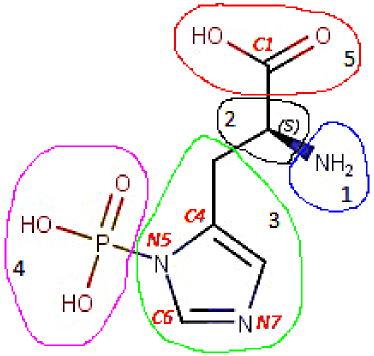	N[C@@H](CC1=CN=C[N]1P(=O)(O)O)C(=O)O	15458487
3-Phosphono-D-histidine;<br>3-phospho-D-histidine	h[5*]	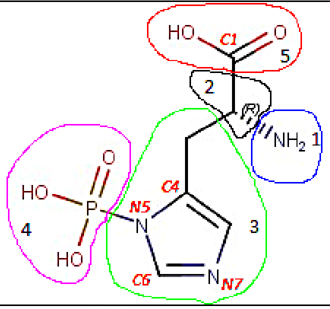	N[C@H](CC1=CN=C[N]1P(=O)(O)O)C(=O)O	-
N1-phosho-L-tryptophan<br>N1-phoshotryptophan	W[6*]	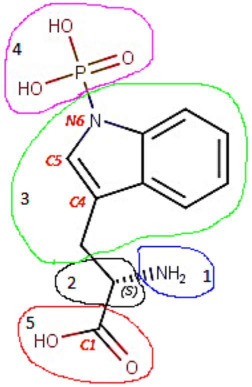	N[C@@H](CC1=C[N](P(=O)(O)O)C2=CC=CC=C12)C(=O)O	14102149

	w[6*]	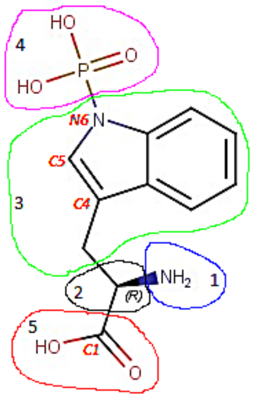	N[C@H](CC1=C[N](P(=O)(O)O)C2=CC=CC=C12)C(=O)O	-
S-phosphono-L-cysteine;<br>S-phosphocysteine	C[3*]	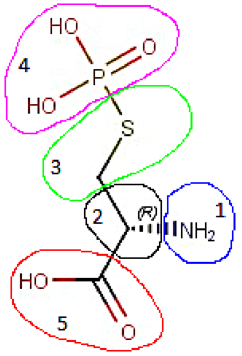	N[C@@H](CSP(=O)(O)O)C(=O)O	3082729
S-phosphono-D-cysteine;<br>S-phospho-D-cysteine	c[3*]	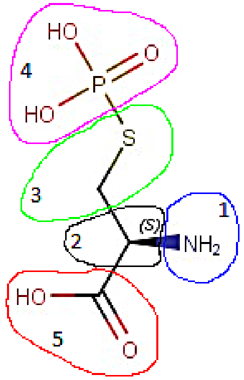	N[C@H](CSP(=O)(O)O)C(=O)O	90158551

L-serine phosphoester	S[1*]	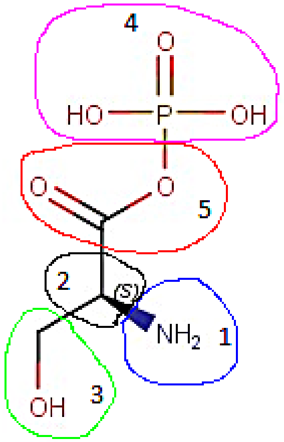	N[C@@H](CO)C(=O)OP(=O)(O)O	11830115
2-phosphonoamino-L-serine	S[2*]	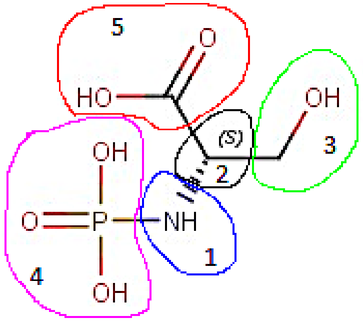	N(P(=O)(O)O)[C@@H](CO)C(=O)O	20057082
Diphospho-L-serine	S[3**]	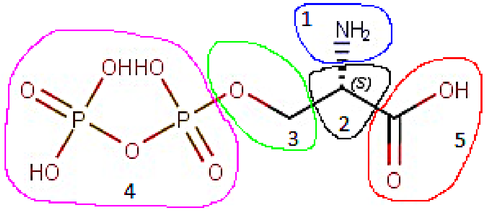	N[C@@H](COP(=O)(O)OP(=O)(O)O)C(=O)O	22865313

Triphospho-L-serine	S[3***]	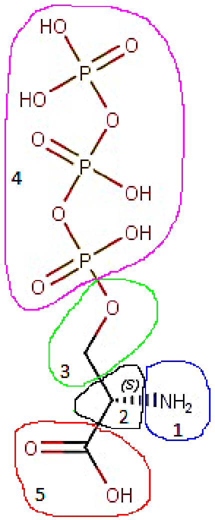	N[C@@H](COP(=O)(O)OP(=O)(O)OP(=O)(O)O)C(=O)O	129891994
3-aminephosphoryl-L-serine	S[3*~]	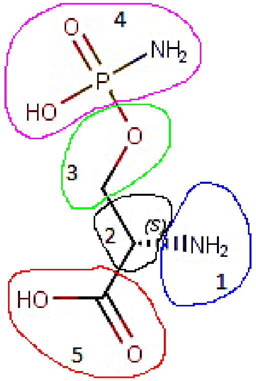	N[C@@H](COP(=O)(O)N)C(=O)O	49867695

3-diaminephosphoryl-L-serine	S[3*[~]~]	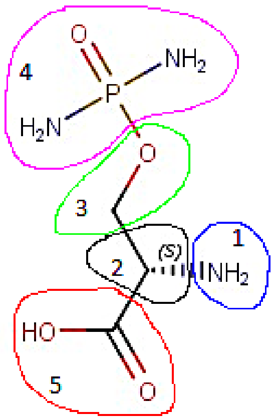	N[C@@H](COP(=O)(N)N)C(=O)O	140086448
(2S)-2-amino-3-[({[amino(hydroxy)phosphoryl]oxy}(hydroxy)phosphoryl)oxy]propanoic acid	S[3**~]	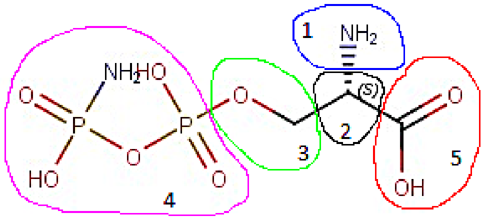	N[C@@H](COP(=O)(O)OP(=O)(O)N)C(=O)O	-
(2S)-2-amino-3-{[hydroxy(phosphonoamino)phosphoryl]oxy}propanoic acid	S[3*~*]	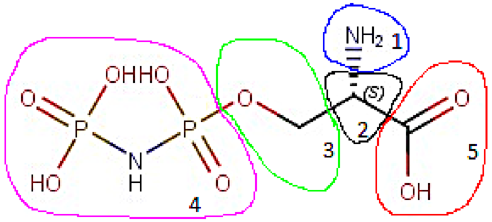	N[C@@H](COP(=O)(O)NP(=O)(O)O)C(=O)O	-
(2S)-2-amino-3-{[amino(phosphonooxy)phosphoryl]oxy}propanoic acid	S[3*[~]*]	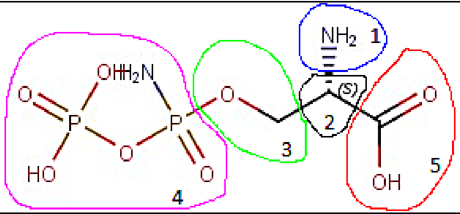	N[C@@H](COP(=O)(N)OP(=O)(O)O)C(=O)O	-

{[(2S)-2-amino-3-(phosphonooxy)propanoyl]oxy}phosphonic acid	S[1*][3*]	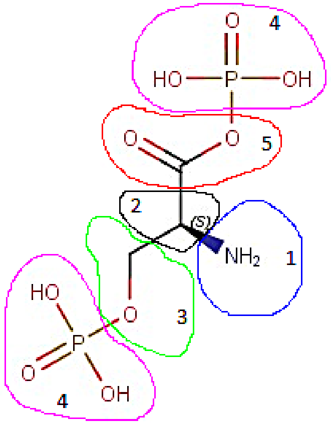	N[C@@H](COP(=O)(O)O)C(=O)OP(=O)(O)O	-
[(2S)-2-amino-2-carbamoylethoxy]phosphonic acid	S~[3*]	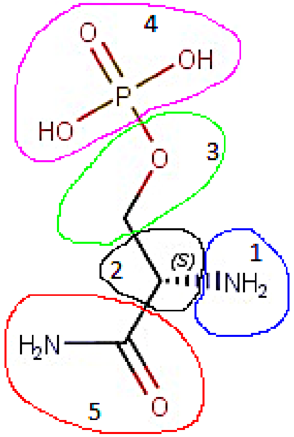	N[C@@H](COP(=O)(O)O)C(=O)N	24892820

[1] Labels and colors used in structures and SMILES strings: α-amine group – 1, blue; α-carbon atom – 2, black; side chain – 3; green; phosphate group – 4, pink; α-carboxyl group – 5, red, according to the convention used by Minkiewicz et al. [65]. Figures prepared using MarvinSketch software

[2] SMILES representations are arranged according to the rules of CHUCKLES algorithm [66], utilized in bioinformatic tools [25,34,36,39,65]

[3] SMILES representations of aromatic amino acids are written using "Kekule" version, recommended for search engines and used in PubChem database [67]

[4] SMILES string arranged manually

Table S2. Examples of recognition patterns associated with attachment points for phosphate groups in amino acid residues.

Amino acid	SMILES representation	Phosphorylation site	Annotation of phosphorylation site	Recognition pattern	Pattern to be inserted	Location relative to recognition pattern
Serine	N[C@@H](CO)C(=O)O	C1	S[1*]	C(=O)O	P(=O)(O)O	After
		C2	S[2*]	N	(P(=O)(O)O)	After
		C3	S[3*]	[C@@H](CO	P(=O)(O)O	After
D-serine	N[C@H](CO)C(=O)O	C1	s[1*]	C(=O)O	P(=O)(O)O	After
		C2	s[2*]	N	(P(=O)(O)O)	After
		C3	s[3*]	[C@H](CO	P(=O)(O)O	After
Threonine	N[C@@H]([C@H](O)C)C(=O)O	C1	T[1*]	C(=O)O	P(=O)(O)O	After
		C2	T[2*]	N	(P(=O)(O)O)	After
		C3	T[3*]	([C@H](O	P(=O)(O)O	After
D-Threonine	N[C@H]([C@@H](O)C)C(=O)O	C1	t[1*]	C(=O)O	P(=O)(O)O	After
		C2	t[2*]	N	(P(=O)(O)O)	After
		C3	t[3*]	([C@@H](O	P(=O)(O)O	After
Tyrosine	N[C@@H](CC1=CC=C(C=C1)O)C(=O)O	C1	Y[1*]	C(=O)O	P(=O)(O)O	After
		C2	Y[2*]	N	(P(=O)(O)O)	After
		C7	Y[7*]	(CC1=CC=C(C=C1)O	P(=O)(O)O	After
D-Tyrosine	N[C@H](CC1=CC=C(C=C1)O)C(=O)O	C1	y[1*]	C(=O)O	P(=O)(O)O	After
		C2	y[2*]	N	(P(=O)(O)O)	After

		C7	y[7*]	(CC1=CC=C(C=C1)O	P(=O)(O)O	After
3-Hydroxyproline	N1[C@@H]([C@@H](O)CC1)C(=O)O	C1	<Hyp3(S)>[1*]	C(=O)O	P(=O)(O)O	After
		C2	<Hyp3(S)>[2*]	N1	(P(=O)(O)O)	After
		C3	<Hyp3(S)>[3*]	[C@@H](O	P(=O)(O)O	After
3-D-Hydroxyproline	N1[C@H]([C@H](O)CC1)C(=O)O	C1	<D-Hyp3(R)>[1*]	C(=O)O	P(=O)(O)O	After
		C2	<D-Hyp3(R)>[2*]	N1	(P(=O)(O)O)	After
		C3	<D-Hyp3(R)>[3*]	[C@H](O	P(=O)(O)O	After
4-Hydroxyproline	N1[C@@H](C[C@@H](O)C1)C(=O)O	C1	<Hyp4(R)>[1*]	C(=O)O	P(=O)(O)O	After
		C2	<Hyp4(R)>[2*]	N1	(P(=O)(O)O)	After
		C4	<Hyp4(R)>[4*]	[C@@H](O	P(=O)(O)O	After
4-D-Hydroxyproline	N1[C@H](C[C@H](O)C1)C(=O)O	C1	<D-Hyp4(S)>[1*]	C(=O)O	P(=O)(O)O	After
		C2	<D-Hyp4(S)>[2*]	N1	(P(=O)(O)O)	After
		C4	<D-Hyp4(S)>[4*]	[C@H](O	P(=O)(O)O	After
5-Hydroxylysine	N[C@@H](CC[C@@H](O)CN)C(=O)O	C1	<Hyl5(R)>[1*]	C(=O)O	P(=O)(O)O	After
		C2	<Hyl5(R)>[2*]	[C@@H](C	(P(=O)(O)O)	Before
		C5	<Hyl5(R)>[5*]	[C@@H](O	P(=O)(O)O	After
		C6	<Hyl5(R)>[6*]	(O)CN	P(=O)(O)O	After
5-D-Hydroxylysine	N[C@H](CC[C@H](O)CN)C(=O)O	C1	<D-Hyl5(S)>[1*]	C(=O)O	P(=O)(O)O	After
		C2	<D-Hyl5(S)>[2*]	[C@H](C	(P(=O)(O)O)	Before
		C5	<D-Hyl5(S)>[5*]	[C@H](O	P(=O)(O)O	After

		C6	<D-Hyl5(S)>[6*]	(O)CN	P(=O)(O)O	After
Aspartic acid	N[C@@H](CC(=O)O)C(=O)O	C1	D[1*]	O)C(=O)O	P(=O)(O)O	After
		C2	D[2*]	N	(P(=O)(O)O)	After
		C4	D[4*]	CC(=O)O	P(=O)(O)O	After
D-Aspartic acid	N[C@H](CC(=O)O)C(=O)O	C1	d[1*]	O)C(=O)O	P(=O)(O)O	After
		C2	d[2*]	N	(P(=O)(O)O)	After
		C4	d[4*]	CC(=O)O	P(=O)(O)O	After
Glutamic acid	N[C@@H](CCC(=O)O)C(=O)O	C1	E[1*]	O)C(=O)O	P(=O)(O)O	After
		C2	E[2*]	N	(P(=O)(O)O)	After
		C5	E[5*]	CC(=O)O	P(=O)(O)O	After
D-Glutamic Acid	N[C@H](CCC(=O)O)C(=O)O	C1	e[1*]	O)C(=O)O	P(=O)(O)O	After
		C2	e[2*]	N	(P(=O)(O)O)	After
		C5	e[5*]	CC(=O)O	P(=O)(O)O	After
Lysine	N[C@@H](CCCCN)C(=O)O	C1	K[1*]	C(=O)O	P(=O)(O)O	After
		C2	K[2*]	[C@@H]	(P(=O)(O)O)	Before
		C6	K[6*]	CN	P(=O)(O)O	After
D-Lysine	N[C@H](CCCCN)C(=O)O	C1	k[1*]	C(=O)O	P(=O)(O)O	After
		C2	k[2*]	[C@H]	(P(=O)(O)O)	Before
		C6	k[6*]	CN	P(=O)(O)O	After
Arginine	N[C@@H](CCCNC(=N)N)C(=O)O	C1	R[1*]	C(=O)O	P(=O)(O)O	After

		C2	R[2*]	[C@@H]	(P(=O)(O)O)	Before
		C6	R[7*]	(=N)N	P(=O)(O)O	After
D-Arginine	N[C@H](CCCNC(=N)N)C(=O)O	C1	r[1*]	C(=O)O	P(=O)(O)O	After
		C2	r[2*]	[C@H]	(P(=O)(O)O)	Before
		C6	r[7*]	(=N)N	P(=O)(O)O	After
Histidine	N[C@@H](CC1=CN=C[NH]1)C(=O)O	C1	H[1*]	C(=O)O	P(=O)(O)O	After
		C2	H[2*]	[C@@H]	(P(=O)(O)O)	Before
		N5	H[5*]	C1=CN=C[NH]1	C1=CN=C[N]1P(=O)(O)O	Instead of
		N7	H[7*]	C1=CN=C[NH]1	C1=C[N](C=N1)P(=O)(O)O	Instead of
D-Histidine	N[C@@H](CC1=CN=C[NH]1)C(=O)O	C1	h[1*]	C(=O)O	P(=O)(O)O	After
		C2	h[2*]	[C@H]	(P(=O)(O)O)	Before
		N5	h[5*]	C1=CN=C[NH]1	C1=CN=C[N]1P(=O)(O)O	Instead of
		N7	h[7*]	C1=CN=C[NH]1	C1=C[N](C=N1)P(=O)(O)O	Instead of
Tryptophan	N[C@@H](CC1=C[NH]C2=CC=CC=C12)C(=O)O	C1	W[1*]	C(=O)O	P(=O)(O)O	After
		C2	W[2*]	[C@@H]	(P(=O)(O)O)	Before
		N6	W[6*]	(CC1=C[NH]	(CC1=C[N](P(=O)(O)O)	Instead of
D-tryptophan	N[C@H](CC1=C[NH]C2=CC=CC=C12)C(=O)O	C1	w[1*]	C(=O)O	P(=O)(O)O	After
		C2	w[2*]	[C@H]	(P(=O)(O)O)	Before
		N6	w[6*]	(CC1=C[NH]	(CC1=C[N](P(=O)(O)O)	Instead of

Color code the same as in Table S1.

Table S3. Examples of the insertion of modifications within phosphate groups

Substrate residue	Product residue	SMILES representation of substrate	Modification site	Annotation of modification site	Recognition pattern	Pattern to be inserted	Location relative to recognition pattern
O-phospho-L-serine	Diphospho-L-serine	N[C@@H](COP(=O)(O)O)C(=O)O	P5	S[3**]	P(=O)(O)O	P(=O)(O)O	After
	3-aminephosphoryl-L-serine		P5	S[3*~]	P(=O)(O)O	P(=O)(O)N	Instead of
	3-diaminephosphoryl-L-serine		P5	S[3*[~]~]	P(=O)(O)O	P(=O)(N)N	Instead of
Diphospho-L-serine	Triphospho-L-serine	N[C@@H](COP(=O)(O)OP(=O)(O)O)C(=O)O	P7	S[3***]	P(=O)(O)OP(=O)(O)O	P(=O)(O)O	After
	(2S)-2-amino-3-[({[amino(hydroxy)phosphoryl]oxy}(hydroxy)phosphoryl)oxy]propanoic acid		P7	S[3**~]	P(=O)(O)OP(=O)(O)O	P(=O)(O)OP(=O)(O)N	Instead of
	(2S)-2-amino-3-{[amino(phosphonooxy)phosphoryl]oxy}propanoic acid		P5	S[3*[~]*]	P(=O)(O)OP(=O)(O)O	P(=O)(N)OP(=O)(O)O	Instead of
3-aminephosphoryl-L-serine	(2S)-2-amino-3-{[hydroxy(phosphonoamino)phosphoryl]oxy}propanoic acid	N[C@@H](COP(=O)(O)N)C(=O)O	P5	S[3*~*]	P(=O)(O)N	P(=O)(O)O	After

For interpretation of the colors, see the explanation provided in Table S1

a: N[C@@H]CC(=O)ON[C@@](COP(=O)(O)O)N[C@@H]([C@H](O)C)C(=O)ON[C@@H]CC(=O)OP(=O)(O)O

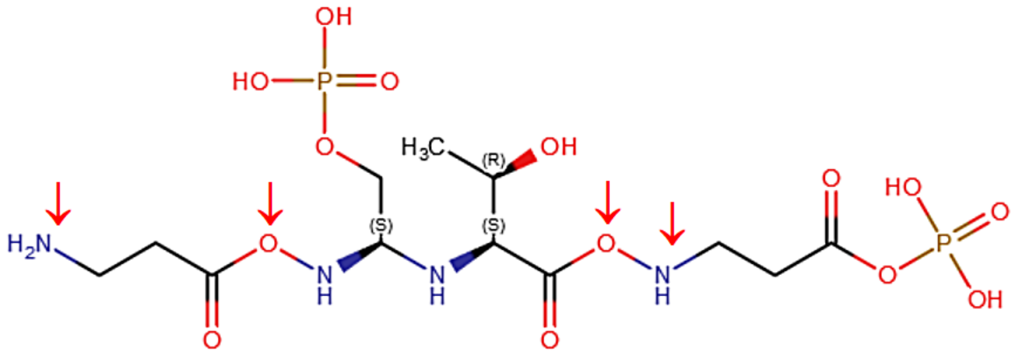

b: N[C@@H](C)C(=O)N[C@@](COP(=O)(O)O)N[C@@H]([C@H](O)C)C(=O)N[C@@]([H])(C)C(=O)OP(=O)(O)O

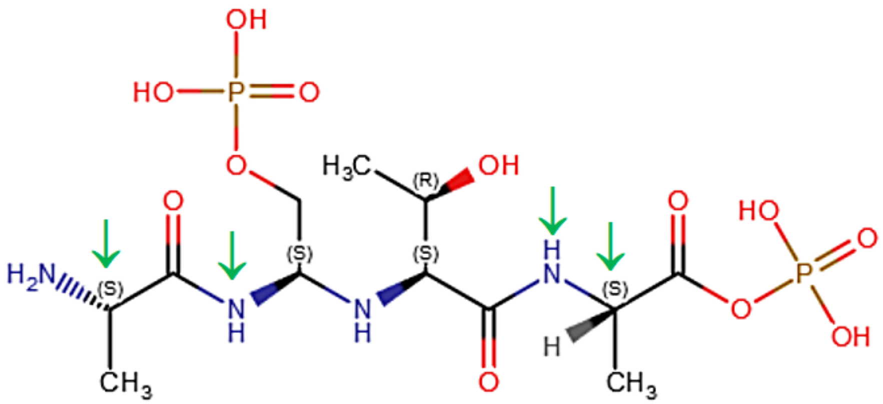

Figure S1. SMILES representations of peptide **D<Hyp3(S)>[6**~]H[7*]** and corresponding strucure. a: Incorrect structures, errors are indicated using red font and red arrows; b: Corrected structure: corrected errors are indicated using green font and green arrows.

a: N[C@@H](CC(=O)O)C(=O)**ON**1[C@@H]([C@@H](OP(=O)(O)OP(=O)(O)N)CC1)C(=O)**ON**[C@@H](CC1=C[N](C=N1)P(=O)(O)O)C(=O)O

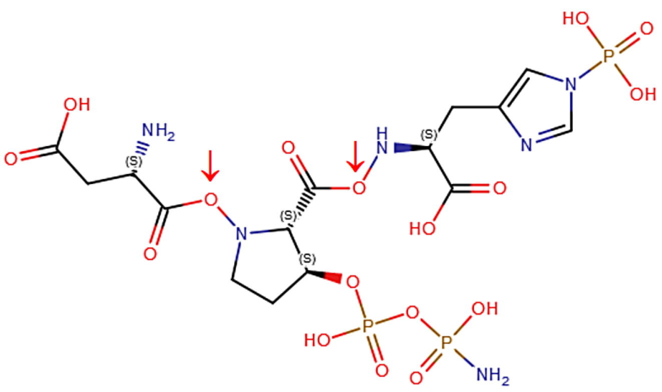

b: N[C@@H](CC(=O)O)C(=O)**N**1[C@@H]([C@@H](OP(=O)(O)OP(=O)(O)N)CC1)C(=O)**N**[C@@H](CC1=C[N](C=N1)P(=O)(O)O)C(=O)O

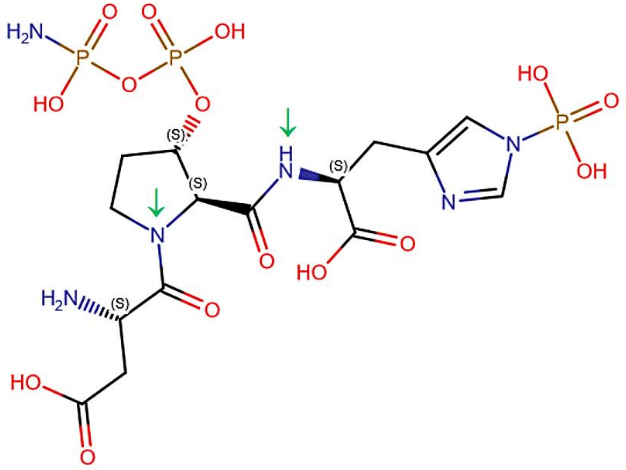

Figure S2. SMILES representation of peptide **GW[6*~*]E[1*]** and corresponding strucure. a: Incorrect structure, errors are indicated using red font and red arrows; b: Corrected structure: corrected errors are indicated using green font and green arrows.

a: NCC(=O)ON[C@@](CC1=C[N](P(=O)(O)NP(=O)(O)O)C2=CC=CC=C12)C(=O)ON[C@@H]CCC(=O)C(=O)OP(=O)(O)O

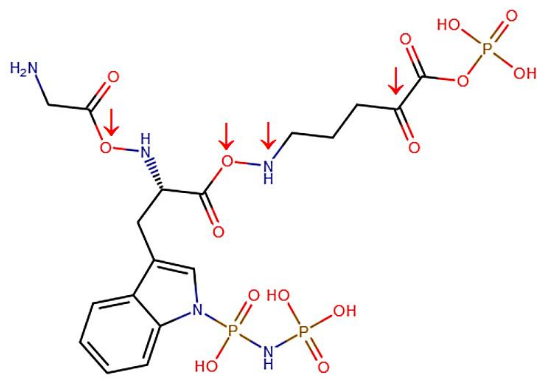

b: NCC(=O)N[C@@](CC1=C[N](P(=O)(O)NP(=O)(O)O)C2=CC=CC=C12)C(=O)N[C@@]([H])(CCC(=O)O)C(=O)OP(=O)(O)O

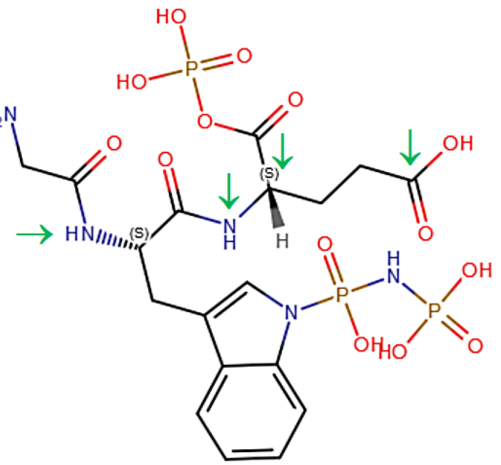

Figure S3. SMILES representation of peptide **AS[3*]TA[1*]** and corresponding strucure. a: Incorrect structure, errors are indicated using red font and red arrows; b: Corrected structure: corrected errors are indicated using green font and green arrows.

a:

N(P(=O)(O)N)[C@@H](CCCCN)C(=O)**ON(P(=O)(O)N)**[C@@H](CCCCNP(=O)(O)O)C(=O)**ON**[C@@H](**C**CC(=O)O)C(=O)**ON**[C@@H]**C**C(=O)**ON**[C@H](CCC(=O)O)C(=O)**ON**[C@H]**CC1=CC=C(C=C1)**C(=O)O

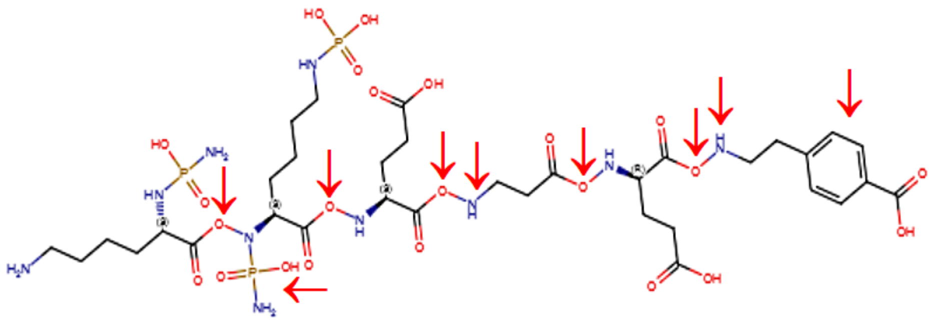

b:

N(P(=O)(O)N)[C@@H](CCCCN)C(=O)**N**[C@@H](CCCCNP(=O)(O)O)C(=O)**N**[C@@H](CCC(=O)O)C(=O)**N**[C@@H]**(C)**C(=O)**N**[C@H](CCC(=O)O)C(=O)**N**[C@H]**(CC1=CC=C(C=C1))**C(=O)O

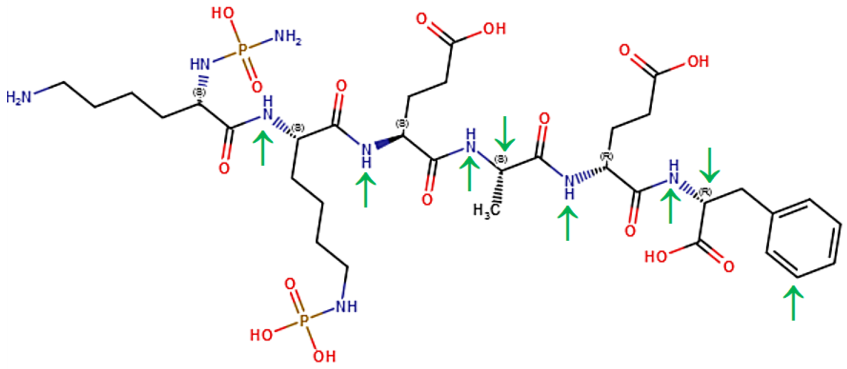

Figure S4. SMILES representation of peptide **K[2*~]K[6*]EAef** and corresponding strucure. a: Incorrect structures, errors are indicated using red font and red arrows; b: Corrected structure: corrected errors are indicated using green font and green arrows.

a: N[C@@H](CC(=O)O)C(=O)ON[C@@H](CC[C@@H](OP(=O)(O)O)CNP(=O)(O)O)C(=O)ON[C@@H](CCCNC(=N)N)C(=O)ON[C@H](CCCNC(=N)N)C(=O)O

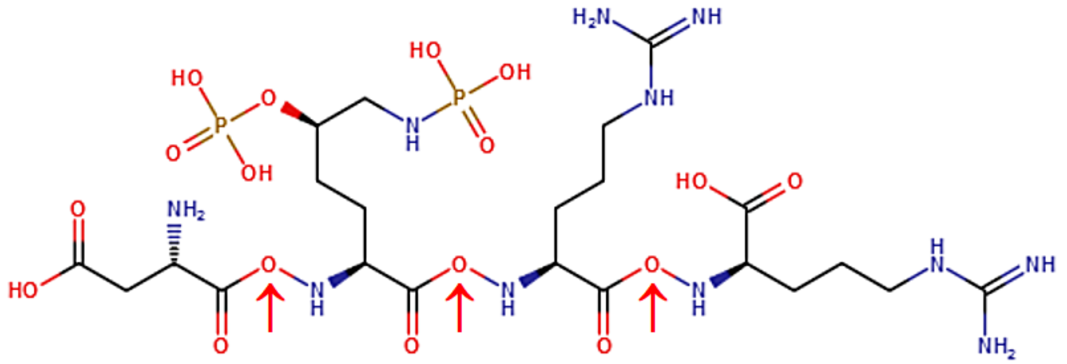

b: N[C@@H](CC(=O)O)C(=O)N[C@@H](CC[C@@H](OP(=O)(O)O)CNP(=O)(O)O)C(=O)N[C@@H](CCCNC(=N)N)C(=O)N[C@H](CCCNC(=N)N)C(=O)O

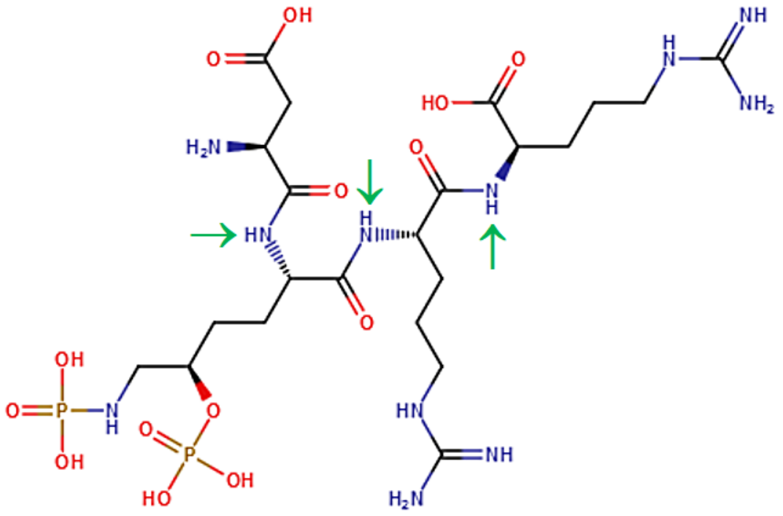

Figure S5. SMILES representation of peptide **D<Hyl5(R)>[5*][6*]Rr** and corresponding strucure. a: Incorrect structures, errors are indicated using red font and red arrows; b: Corrected structure: corrected errors are indicated using green font and green arrows.
